# Heterocycle-Based Multicomponent Reactions in Drug Discovery: From Hit Finding to Rational Design

**DOI:** 10.3390/biomedicines10071488

**Published:** 2022-06-23

**Authors:** Pau Nadal Rodríguez, Ouldouz Ghashghaei, Andrea Bagán, Carmen Escolano, Rodolfo Lavilla

**Affiliations:** Laboratory of Medicinal Chemistry, Faculty of Pharmacy and Food Sciences, University of Barcelona and Institute of Biomedicine (IBUB), Avda Joan XXIII 27–30, 08028 Barcelona, Spain; pnadal@ub.edu (P.N.R.); ghashghaei@ub.edu (O.G.); abaganpolonio@ub.edu (A.B.)

**Keywords:** bioprobes, drug discovery, heterocycles, multicomponent reactions, reaction discovery

## Abstract

In the context of the structural complexity necessary for a molecule to selectively display a therapeutical action and the requirements for suitable pharmacokinetics, a robust synthetic approach is essential. Typically, thousands of relatively similar compounds should be prepared along the drug discovery process. In this respect, heterocycle-based multicomponent reactions offer advantages over traditional stepwise sequences in terms of synthetic economy, as well as the fast access to chemsets to study the structure activity relationships, the fine tuning of properties, and the preparation of larger amounts for preclinical phases. In this account, we briefly summarize the scientific methodology backing the research line followed by the group. We comment on the main results, clustered according to the targets and, finally, in the conclusion section, we offer a general appraisal of the situation and some perspectives regarding future directions in academic and private research.

## 1. Introduction

Modern drug discovery is a complex endeavor where pharmacology, biology and medicinal chemistry play important roles. Yet it unavoidably relies on a pivotal organic synthesis basis to ensure the access to the desired molecules. It has been estimated that thousands of new compounds are needed to launch a drug. This synthetic activity pervades all phases in drug development from the hit finding to production, and lately has experienced impressive advances [1,2,3]. Although all stages have specific requirements and are amenable to improvement, we believe that the synthetic methodology regarding aspects such as bond forming efficiency, structural diversity, and reaction economies are crucial points in this issue, the time factor being especially relevant [4,5,6,7].

The concern about the relevant chemical space in a given MedChem project, although capital, is often overlooked [8,9], considering that the dimension of drug-like space is around 10^60^ virtual molecules and, likely, we should go beyond this limit to find new medicines [10]. This situation can be confronted with the fact that at present, we have access to more than a 100 billion compounds, and the trillion level is in sight [11]. Furthermore, the broadcasted compounds are accessible through a limited set of well-established reactions, around 20 processes dominating the stepwise preparations in medicinal chemistry [12]. This only represents a minute fraction of the in principle feasible chemical connectivities, with most accessible scaffolds being overexploited. Thus, finding relevant drugs should arguably rely on the incorporation of new scaffolds, rather than overcrowding the existing ones [13].

To tackle the aforementioned problems, we became interested in multicomponent reactions (MCRs), processes where three or more reactants interact to form an adduct with most atoms of the starting materials, in one step, through a domino-type transformation involving a unified mechanism [14,15]. The development of MCRs continues at a considerable pace in reaction discovery, organic synthesis, etc., and their application in medicinal chemistry is gaining momentum. The implementation of this methodology is having a deep impact on hit finding, greatly helping the production of combinatorial libraries (key for SAR studies), hit-to-lead (H2L) stage, lead optimization and large-scale preparation of complex medicines [16,17,18].

**Figure 1:**
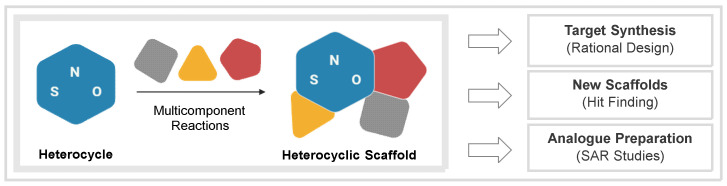
Heterocycle-based MCRs for MedChem applications.

Our group has been involved in the development of new MCRs based on the use of fundamental heterocycles as substrates. The following reasons may justify this approach: (a) the limited knowledge available on these processes; (b) the presence of heterocyclic motifs in the majority of drugs and (c) the convenience of the MCR approach to tackle defined MedChem projects, both from a conceptual and practical point of view (Figure 1) [19,20,21,22]. In this review, we summarize the biomedical impact of our research, classified according to the biological targets upon which the MCR adducts interact, their mode of action and their purpose. The following sections comprise enzymes, transcription factors, chemotherapeutics, fluorescent probes, and neuroactive compounds. Finally, some considerations will be disclosed as conclusions and future outlook.

## 2. Enzymes

### 2.1. Acetyl-Cholinesterase (AChE)

**Figure 2:**
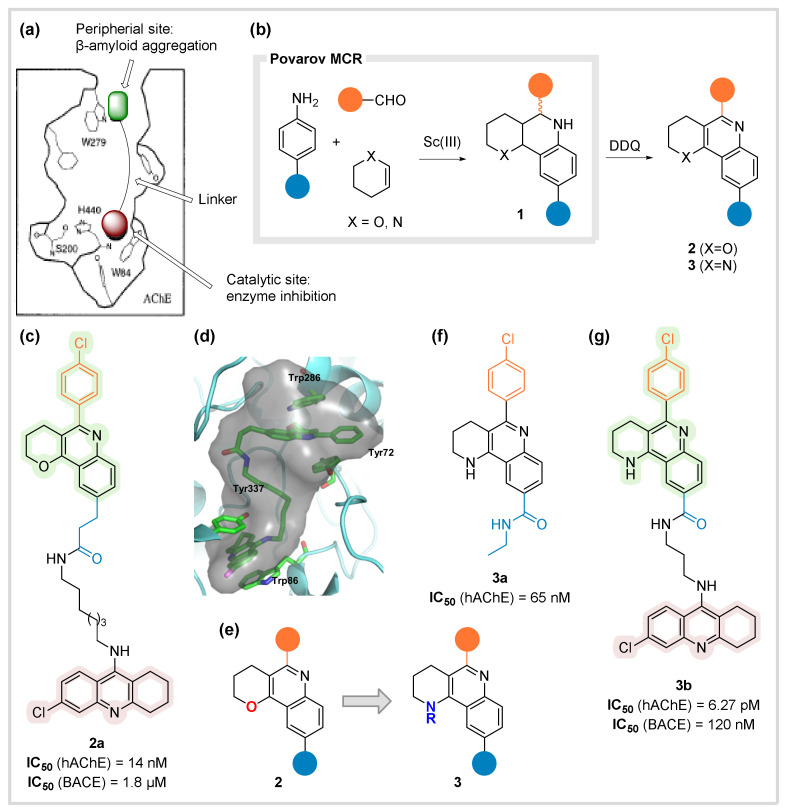
AChE inhibitors. (**a**) Outline of the AChE dual inhibition. (**b**) The Povarov MCR and subsequent oxidation leading to the new peripheral site inhibitors. (**c**) Structure and pharmacological data for the dual AChE inhibitor **2a**. (**d**) Docking simulation showing the interactions at the catalytic and peripheral sites. From ref. [28] (P. Camps et al., *J. Med. Chem*. **2009**, *52*, 5365), reproduced with permission. Copyright 2009 ACS. (**e**) Isosteric modification in the peripheral site inhibitor core. (**f**) Structure and pharmacological data of small molecule peripheral site inhibitor **3a**. (**g**) Structure and pharmacological data for the dual AChE inhibitor **3b**.

In this section, we account for the research targeting the dual inhibition of the acetylcholinesterase (AChE). Although blocking the enzyme improves the symptoms of Alzheimer disease (AD), Inestrosa et al. demonstrated that the enzyme itself promotes amyloid peptide aggregation, in vitro and in vivo, through binding at a peripheral site [23]. At this point, P. Camps and D. Muñoz-Torrero (UB), who had prepared novel classes of extremely potent AChE inhibitors [24], contacted us to address a challenging task: the dual inhibition of AChE at the active and peripheral sites [25]. Since the putative binding site of the amyloid peptide at the enzyme surface is quite flat, the plan was to drive the inhibitor into the peripheral site, while efficiently blocking the catalytic site with a known blocker, a tacrine derived unit, both linked by a flexible chain to fill the gorge that communicates both sites (Figure 2a). Preliminary docking studies backed the design of the final compounds. As the peripheral site binder, a planar pyrano-quinoline nucleus was chosen and the retrosynthesis relied on a Povarov MCR [26], a process which is progressively used in medicinal chemistry [27]. In this way, the condensation of an aniline, an aromatic aldehyde, and an activated olefin afforded the corresponding MCR adducts **1** as a mixture of stereoisomers, which were subjected to DDQ oxidation to afford the fused aromatic systems (Figure 2b). The resulting fragment was coupled with an amino-chlorotacrine to yield the designed compound **2a** in a remarkable three-step preparation (Figure 2c). Compound **2a** exhibited impressive performance, displaying potent central inhibition at nanomolar level, together with spontaneous and AChE-mediated inhibition of the amyloid aggregation. Interestingly, it showed a suitable PAMPA profile, as well as an unexpected BACE inhibition (Figure 2c) [28]. Refined computational calculations, done by F. J. Luque (UB), showed a favored pose of the dual inhibitor blocking the active site with its tacrine moiety, filling the gorge with the flexible chain, and nicely disposing the MCR-derived unit at the peripheral site, sandwiched between a tyrosine and a tryptophan (Figure 2d).

Careful analysis of the observed interactions suggested a structural change to maximize the binding at the peripheral site which has an anionic nature: to isosterically replace the oxygen atom at the pyran ring with a nitrogen. This would ensure the amine protonation at physiological pH, reinforcing the affinity with the aromatic amino acid residues involved by adding a cation-π interaction (Figure 2e). In this way, a related Povarov MCR with tetrahydropyridines conveniently afforded the desired adducts, only requiring tactical modifications such as the introduction of substitution at the nitrogen in the precursors or the use of a Boc group to generate the final NH derivatives after deprotection. In this way, we prepared a chemset of exclusively allosteric binders with potency levels reaching low nanomolar range, a remarkable result for small molecules tackling the peripheral site (**3a**, Figure 2f) [29]. The mode of action is an allosteric inhibition, in agreement with enzymatic data and supported by molecular modeling studies. Furthermore, following this research line, a dual inhibitor was envisaged, linking this peripheral unit to the chlorotacrine moiety. Docking studies suggested an attractive interaction of the amide group with an amino acid residue at the gorge, resulting in a deeper penetration of the quinoline substructure (in comparison with dihydropyrane-fused derivatives), recommending a shorter linkage for optimal binding. In this way, derivative **3b** was designed and prepared through a straightforward MCR-based synthesis (Figure 2g). It displayed outstanding potency against AChE with a single digit picomolar activity. Moreover, it showed a good BACE-blocking profile at nanomolar level while remarkably inhibiting the amyloid peptide and tau aggregations [30]. Incidentally, this new class of allosteric binders, having clear analogies with antiparasitic aminoquinolines, were also tested against trypanosomatid causing agents (*Trypanosoma cruzi, T. brucei and Leishmania infantum*), displaying low micromolar levels of IC_50_ and IC_90_, albeit with lesser efficacy in front of epi- and pro-mastigotes and with low safety indexes [31]. In our opinion, the presented results clearly demonstrate how the intelligent implication of the MCR strategy combined with the suitable computational platform can lead to highly efficient production of meaningful chemical libraries and eventual lead compounds. 

### 2.2. Dihydrofolate Reductase (DHFR)

**Figure 3:**
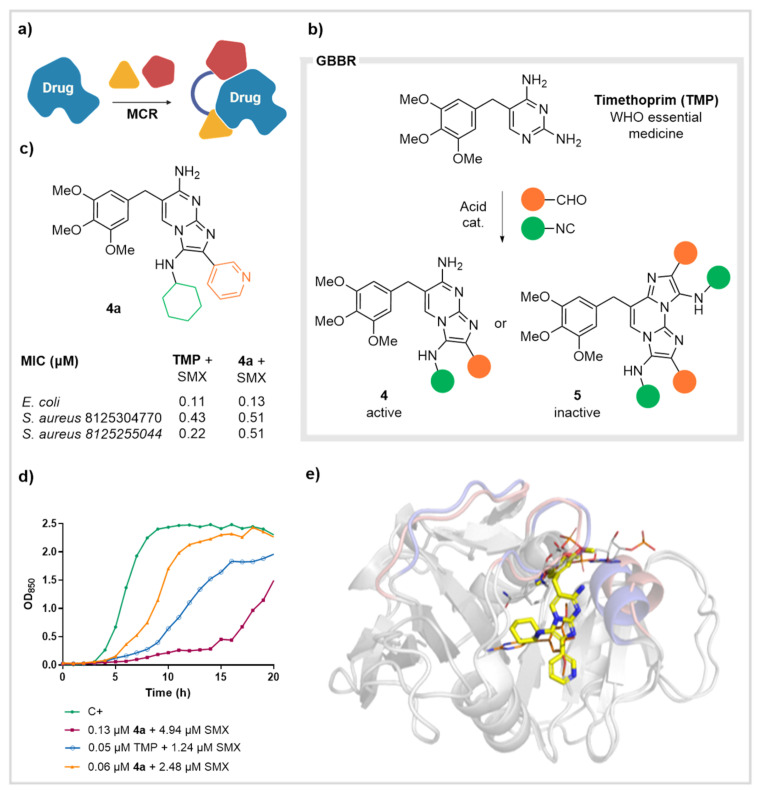
DHFR inhibitors. (**a**) Concept of the *Drugs form Drugs* approach. (**b**) Structure of trimethoprim and GBBR leading to mono- and bis- adducts **4** and **5**, respectively. (**c**) Structure and activity data of lead compound **4a**. (**d**) Growth curve of *E. coli* with TMP and **4a** in combination with SMX. (**e**) Docked position of adduct **4a** (yellow) in DHFR (homology model from *E. coli*). The location of TMP (orange) and NADPH (gray) is also shown.

Another research line in our laboratories aims at the construction of new drugs from existing ones through a sort of late-stage derivatization via MCRs. This *Drugs from Drugs* approach relies on two premises: (a) the existence of drugs that can act as substrates in MCR processes and (b) the suitability of the resulting MCR adducts to keep meaningful structural information and to maintain activity against the initial target (Figure 3a). In this way, we may diversify the original entity and tackle a variety of aspects including drug selectivity, resistance, etc. In this context, MCRs would greatly accelerate the drug discovery process and simultaneously enable serious structural modifications. We focused on the Groebke-Blackburn-Bienaymé MCR (GBBR), the interaction of an aminopyridine, and aldehyde and an isocyanide to yield substituted imidazopyrididine adducts [32,33] knowing that several drugs display a diaminopyrimidine motif. Such is the case of trimethoprim (TMP), a WHO essential medicine for the treatment of lower urinary tract infections and bacterial dysentery. TMP blocks the folic acid route by inhibiting dihydrofolate reductase (DHFR) and is usually used combination with sulfamethoxazole (SMX). The synergistic effect efficiently interrupts DNA synthesis in bacteria (in contrast, humans get the folic acid from diet). Currently, TMP treatments face increasing problems with drug resistant infections, which have become a serious challenge in chemotherapy. Interestingly, TMP was eligible for the MCR-based modification, as the resulting adducts retained a relevant part of the structural binding motif (Figure 3b). Therefore, we tackled the derivatization of TMP to expand the chemical diversity around its core and optimize the activity of the formed derivatives. Accordingly, we promoted selective GBBRs involving TMP, an array of aldehydes and isocyanides to build a representative chemset of 15 TMP mono- and bis-derivatives **4** and **5** (Figure 3b. For further discussion of the exclusive formation of adducts 4 and 5 under mild and harsh conditions respectively, see Section 4.2. 

This collection was tested against *Escherichia coli*, *Pseudomonas aeruginosa* and several strains of methicillin-resistant *Staphylococcus aureus*, to find that double adducts **5** were inactive, whereas derivatives **4** displayed different levels of potency, useful to establish a preliminary SAR. Although *Pseudomonas* were resistant to all new adducts, some of them displayed relevant activity in the low micromolar range. The best hit was derivative **4a**, which, albeit with MIC figures very similar to the parent TMP (Figure 3c), provided a remarkably faster mode of action, a key feature in clinical treatments (Figure 3d) [34]. This was shown in combination experiments with SMX both against *E. coli* and *S. aureus*, prompting new research on its mechanism of action. Additional determinations revealed that the selected compound **4a** was active in biofilm prevention in tests with *P. aeruginosa*, even though similar to TMP, it could not fully eradicate those from *S. aureus*. Furthermore, its toxicity against cell lines (HepG2 and L-929) was extremely low at the usual antibiotic concentrations. *P. aeruginosa* is naturally resistant to TMP and its derivatives, likely due to its efflux systems. Accordingly, treatment with adduct **4a**, SMX and an efflux pump inhibitor (PAβN), enhanced its potency in a quite remarkable manner (15-fold). Similarly, colistin (a peptide used to permeabilize membranes) also exhibits a synergistic effect in resistant bacteria, rendering these microorganisms sensible to TMP-like adducts. Furthermore, in the presence of these inhibitors, the enzyme activity can be fully restored by adding increasing concentration of the NADPH cofactor. To explain this fact and gain knowledge on the mode of action, we explored the binding of MCR adduct **4a** to a model of DHFR from *E. coli*. After refined docking calculations, and comparisons with the PDB from the interaction of the parent TMP with the same enzyme, it was concluded that our compounds present a different binding mode in the pocket. Derivative **4a** is accommodated in a completely rearranged form, sterically colliding with the NADPH cofactor (Figure 3e) [35]. These findings reinforce the need for new scaffolds and validate the hypothesis of using existing drugs to find new relevant derivatives through MCRs.

## 3. Transcription Factors

### Aryl Hydrocarbon Receptor (AhR)

The Aryl hydrocarbon Receptor (AhR) pathway is associated with a vast variety of physiological processes. Interestingly, both activation and inhibition of this pathway result in distinct biological responses. Therefore, its controlled regulation is considered as a potentially promising therapeutic approach for a range of diseases including cancers and immune system disorders. At present, AhR-based drug discovery campaigns face serious challenges. First, as the active site of the receptor is not fully described, the development of novel small-molecule AhR ligands mainly relies on compound screening rather than rational design. On the other hand, the majority of currently known AhR ligands raise cytotoxicity concerns due to their alarming poly(hetero)aromatic structures. Finally, the generation of relevant screening libraries around limited (complex) scaffolds requires multi-step synthetic routes, slowing down the progress in the area.

**Figure 4:**
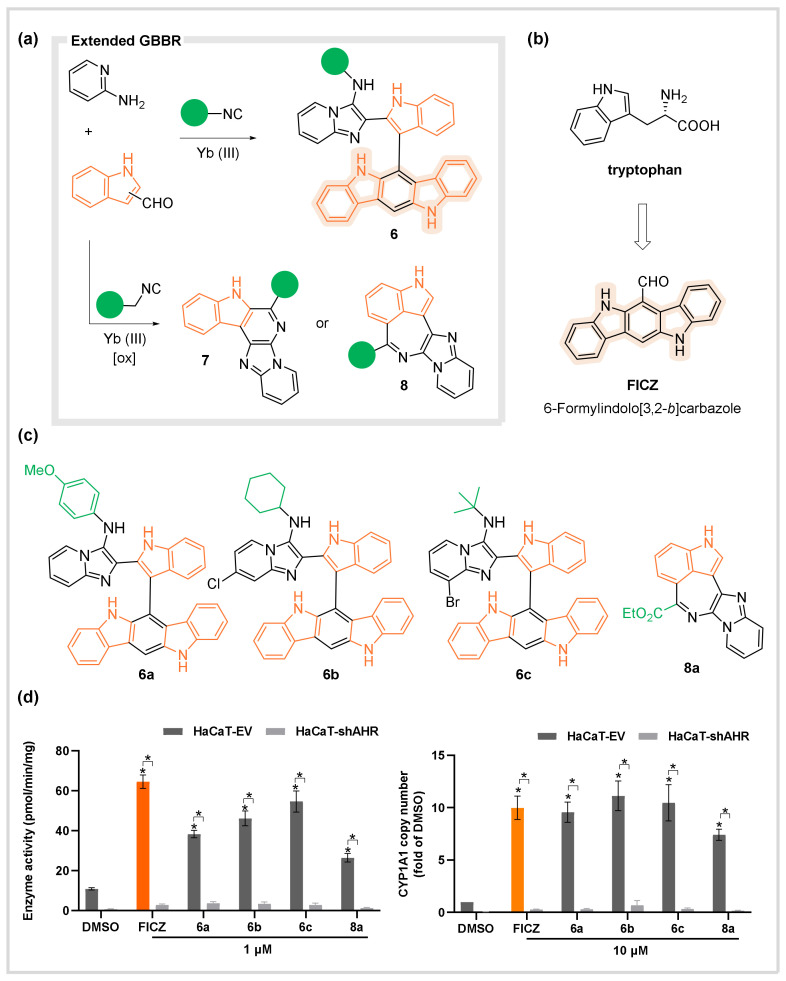
AhR Activators. (**a**) The extended GBBRs with indole carboxaldehydes. (**b**) FICZ ligand structure. (**c**) Novel MCR-based indolocarbazole ligands. (**d**) The AhR-activating properties of the synthesized compounds in keratinocytes (related to cytochrome P450 activity and expression). * *p* ≤ 0.05.

In this context, our group reported an MCR-based pathway to assemble complex fused polyheterocyclic systems through oxidative and non-oxidative extensions of GBBR with indole carboxaldehydes, yielding adducts **6–8** (Figure 4a) [36]. Remarkably, the incorporation of indole 2-carboxaldehydes yielded GBBR adducts **6** featuring indolocarbazole nuclei (Figure 4a). Interestingly, these compounds shared the same structural motif of FICZ, a biosynthetic derivative of tryptophan and a potent activating ligand of AhR, commonly used as a positive control (Figure 4b). Inspired by this resemblance, a selection of synthesized compounds was tested. The results confirmed the substituted indolocarbazoles **6a–c** to be AhR pathway activators (Figure 4c,d). Remarkably, compound **8a**, featuring a complex 7-membered connectivity in a polycyclic framework, demonstrated similar behavior, despite lacking the indolocarbazole residue [36]. Considering the easy access to structurally diverse AhR ligand libraries through one-pot operations, further studies are being conducted to develop novel AhR pathway modulators with potential biomedical applications.

## 4. Chemotherapeutic Agents

Compound screening continues to be a highly influential approach in modern drug discovery. This strategy is particularly useful in case of limited knowledge about the disease development mechanisms, the complexity of the physiological pathways, or insufficient information on the function/structures of the biological targets involved. On such occasions, as computational approaches may not be fully implied, the drug discovery campaigns heavily rely on the screening of relevant libraries. In this context, MCRs are powerful tools for the rapid development of small-molecule collections around promising scaffolds or diversity-oriented motifs [37]. Here, we summarize the applications of the heterocycle-based MCRs discovered in the group to develop novel *tunable* chemotherapeutic agents via compound screening.

### 4.1. Antiparacitic Agents against Trypanosoma

Chagas disease and Sleeping sickness, arising from *Trypanosoma cruzi* and *Trypanosoma brucei*, respectively, figure among the concerning global health problems. These pathologies dramatically affect the lives of large populations and impose considerable economical charge on several countries. Additionally, because of our dynamic lifestyle, they are not geographically restricted to endemic regions anymore. Moreover, as the currently available therapies are not efficient enough, the treatments are long-term, and the patients normally suffer a range of side-effects, experiencing chronic stages and developing resistance. These aspects intensify their socio-economical effects and imply an urgent need for treatments arising from new/modified scaffolds with improved efficiency and safety.

**Figure 5:**
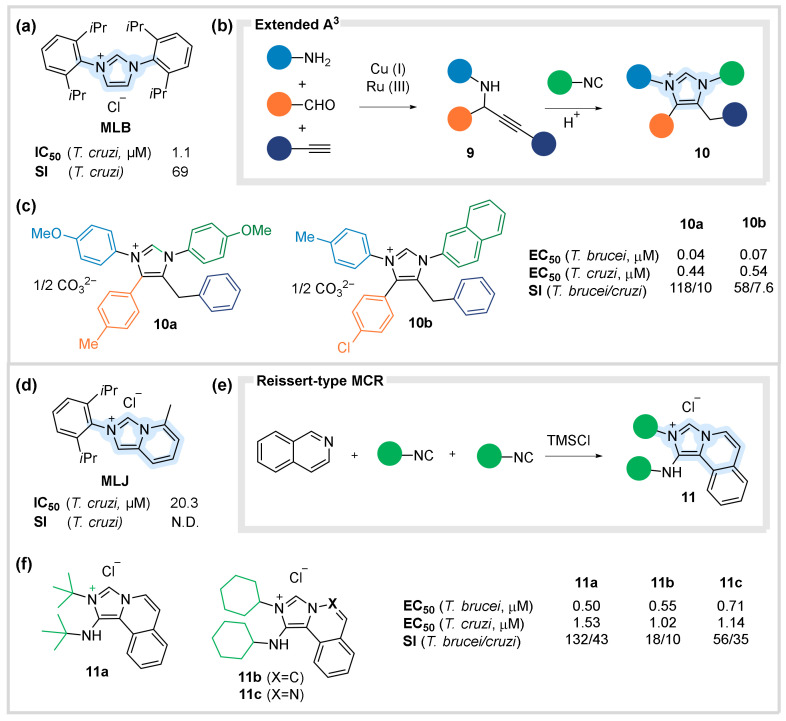
MCR-based antiparasitic agents. (**a**) Imidazolium salt MLB with antiparasitic activity against Chagas disease. (**b**) MCR pathway to tetrasubstituted imidazolium salts. (**c**) Biological activities of promising derivatives. (**d**) Benzimidazolium salt MLJ with mild antiparasitic activity against Chagas disease. (**e**) TMSCl-promoted MCR synthesis of benzimidazolium salts. (**f**) Biological activities of selected derivatives.

In this context, Robello et al. reported the anti-trypanosomal activity of simple imidazolium salts against Chagas disease [38]. Based on their findings, symmetrically N-Substituted imidazolium salts like compound **MLB** showed improved potency and safety against *Trypanosoma cruzi*, compared to the commonly prescribed benznidazole (Figure 5a). In the meantime, our group described the convenient assembly of tetra-substituted imidazolium salts **10** through the acid-catalyzed reaction of propargylamines **9** and isocyanides. The reaction was successfully coupled with the classic A^3^-MCR (Aldehyde-Amine-Alkyne condensation) to prepare the intermediate substrates **9** through the condensations of aldehydes, amines, and alkynes (Figure 5b). The simple (one-pot) preparation and the wide scope of the reaction furnished a small library of imidazolium salts **10** with four diversity points, allowing the incorporation of both aromatic and aliphatic residues [39]. A selection of synthesized compounds was tested against both African and American Trypanosoma. Among them, salts **10a** and **10b** displayed particularly encouraging antiparasitical activity against *Trypanosoma cruzi* at sub-µM level and moderate safety indexes. Surprisingly, these compounds were confirmed to be highly active against *Trypanosoma brucei* (at nanomolar-level) with exceptional safety indexes (Figure 5c) [40].

Interestingly, Robello’s report also suggested the (less potent) antiparasitic activity of a benzimidazolium derivative called **MLJ** (Figure 5d) [38]. Meanwhile, the group was also developing a novel TMSCl-mediated mechanism for the reaction of isocyanides and azines to give amino benzimidazolium salts **11** through a Reissert type MCR (Figure 5e) [41]. This unprecedented mild activation significantly amplified the scope of the reaction, giving access to a diverse collection of aminobenzimidazolium salts. These compounds featured a wide range of aliphatic/aromatic substituents (coming from the isocyanides) as well as variety of azines/diazines. The novelty and features of the synthesized library encouraged us to determine their antiparasitic activity. To our delight, several derivatives including **11a–c** exhibited micromolar-level activity against *Trypanosoma cruzi* with safety indexes comparable to the previous series (Figure 5f). Furthermore, they also confirmed to have remarkable nano-molar potencies and excellent safety indexes against *Trypanosoma brucei.* Having promising pharmacochemical properties, compound **11c** (featuring an added nitrogen atom on the heterocyclic scaffold) was selected for an in vivo study on mice and it was unexpectedly inactive probably due to a poor bioavailability [41]. However, these results confirm the promising potential of scaffolds **10** and **11** as new classes of antiparasitic agents against both African and American Trypanosoma. Moreover, considering the described multicomponent access, their performance and pharmaco-physical properties could be further improved by structural modifications upon the existing diversity points.

### 4.2. Antiviral Agents against Human Adenovirous

Viral diseases (including influenza and related infections) affect large human/animal populations, especially during outbreaks. Moreover, these infections seriously threaten individuals with weakened immune systems. Unfortunately, despite being fatal for high-risk groups, several specific antiviral treatments are not yet available. On the other hand, due to their mechanisms of action, frequent mutations, and developed resistance, there is a constant demand for new scaffolds/derivatives with improved performance.

**Figure 6:**
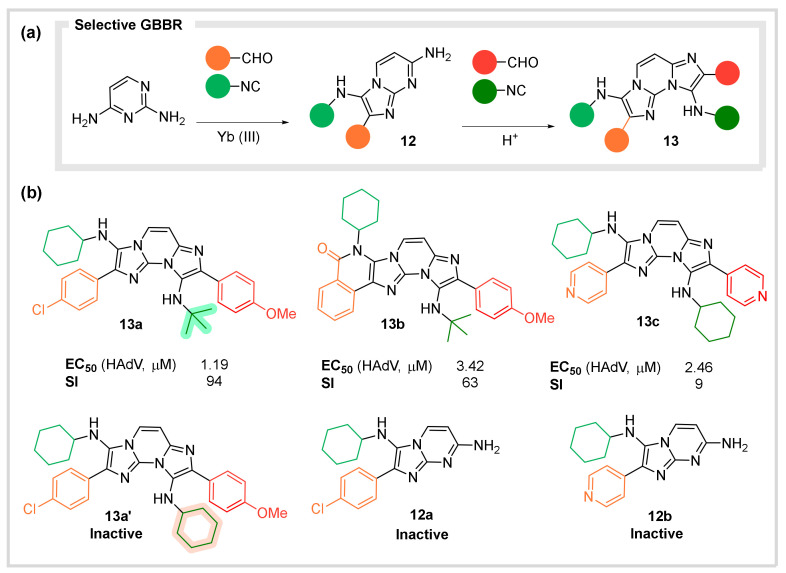
GBBR approach to antivirals. (**a**) Selective GBBRs upon diaminoazines. (**b**) Antiviral activity of the representative GBBR compounds against human Adenovirus.

In this context, a project in the group led to the discovery of multiple GBBRs upon a variety of unexplored aminoazine substrates. The structural diversity, selectivity, scope, and a variety of post-transformations established a unique synthetic platform for screening libraries (Figure 6a) [32]. Inspired by the relevance of the benzimidazole core in medicinal chemistry, especially in anti-infectious compounds, a selection of our GBBR adducts were tested against human adenovirus (Figure 6b). Remarkably, bis-GBBR compounds **13a–c** exhibited micromolar activity, whereas their mono-GBBR precursors **12a–b** were inactive. Surprisingly, compound **13a**′, a close derivative of **13a** with only a slight modification on one isocyanide residue was inactive. Compounds **13a–b** also exhibited remarkable selectivity indexes. We believe that exploiting the structural diversity and synthetic facility of these reactions to further expand the observed SAR may lead to the discovery of specific antiviral agents for human adenovirus and related infections.

## 5. Fluorescent Bioprobes

**Figure 7:**
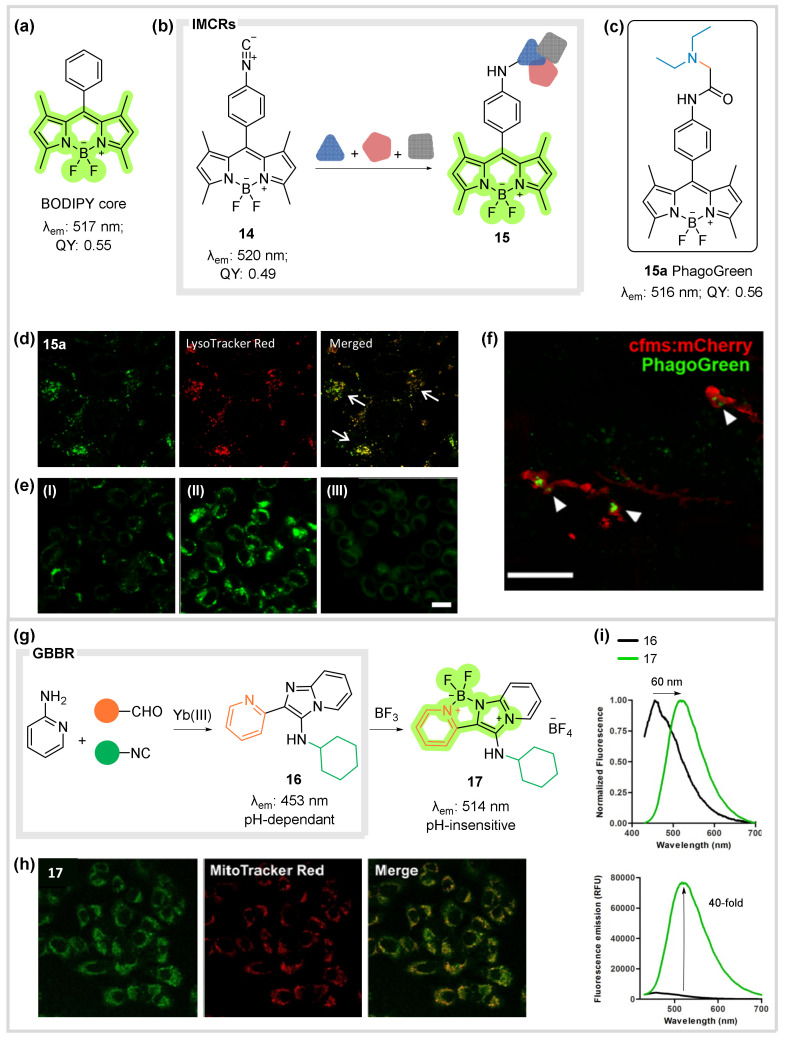
(**a**) Structure and photophysical properties of the BODIPY core. (**b**) Isocyanide derivatized BODIPY scaffold and generation of compounds **15** through various IMCRs. (**c**) Structure and photophysical properties of selected probe **15a**, Phagogreen. (**d**) Co-incubation of **15a** and LysoTracker Red in A549 cells. (**e**) RAW 264.7 macrophages were treated with **15a**. Fluorescence of **15a** in (**I**) non-activated macrophages, (**II**) zymosan-activated macrophages, and (**III**) zymosan-activated macrophages treated with bafilomycin A. (**f**) Still from time-lapse imaging of transgenic zebrafish with m-Cherry-labelled macrophages treated with PhagoGreen. Scale bars: 20 μm. Sections (**d**–**f**) from ref. [47] (O. Vázquez-Romero et al, *J. Am. Chem. Soc*. **2013**, *135*, 16018) reproduced with permission. Copyright 2013 ACS. (**g**) GBBR access to BODIPY-adduct **17**. (**h**) Human A549 epithelial cells upon incubation with compound **17** and MitoTracker Red. Scale bar: 10 µm. (**i**) Fluorescent properties of probe **17** vs. GBBR adduct 16. Sections (**h**,**i**) From ref. [32] (O. Ghashghaei et al., *Chem. Eur. J*. **2018**, *24*, 14513), reproduced with permission Copyright Wiley-VCH GmbH.

The ability of chemical entities to fluoresce upon light excitation has been long exploited in biomedicine and bioimaging [42,43,44]. The development of new fluorescent probes encompasses a compromise between suitable photophysical properties (high fluorescence, quantum yields, etc.) and adequate properties for biological use (good affinity and permeability, low toxicity, etc.). A representative example is the BODIPY scaffold (Figure 7a), one of the most versatile fluorescent probes exploited so far, which plays an extremely important role in bioimaging [45,46]. Taking into account the relevance of isocyanide based MCRs as well as the group’s previous work in the field, we envisioned the participation of a BODIPY derivative in such transformations to rapidly build new fluoroprobes. In this way, we synthesized the unprecedented isonitrile-functionalised BODIPY (14), following a standard preparation protocol form the corresponding anilino-BODIPY derivative. Compound 14 maintained the excellent photophysical properties of the parent BODIPY core (Figure 7b) [47]. Then, isocyanide **14** was used as the substrate in several well-known MCRs, namely Ugi, Passerini and GBBR (Figure 7b) to generate a series of BOPIDY-derivatized analogues **15**, which showed remarkable cell permeability. Imaging experiments were planned and supervised by M. Vendrell (U. Edinburgh). While most derivatives indiscriminately stained the whole cytoplasm, derivative **15a** (pK_a_ ≈ 5.76, Figure 7c) showed selective bright fluorescence in subcellular acidic compartments, as confirmed by co-incubation with acidotropric dye LysoTracker Red (Figure 7d). In light of these remarkable properties, we evaluated the imaging of phagosomal acidification by treating RAW 264.7 macrophages with probe **15a** before and after incubation with zymosan, a glucan that induces phagosomal acidification. It was determined that the fluorescence intensity was proportional to the activation with zymosan (unlike LysoTracker Red), proving that emission of **15a** is ratiometric and depends on macrophages acidification as it decreased upon inhibition of a key ATPase for phagosomal acidification with bafilomycin A (Figure 7e).

We confirmed that compound **15a**, named PhagoGreen, was neither toxic nor altered macrophage biology. Thus, we tackled the in vivo imaging of activated macrophages by incubating zebrafish embryos expressing m-Cherry labelled phagocytic macrophages with PhagoGreen **15a**, and no staining of early phagosomes was observed, whereas bright fluorescence was detected in actively phagocytic macrophages (Figure 7f).

Moreover, we believed that our previous work in MCRs using heterocyclic substrates [22] provided an optimal framework for the development of de novo fluorophores. In this regard, as the group was developing a GBBR-based chemset [32], we envisaged that the input of pyridine-2-carboxaldehyde would lead to generation of compounds resembling the BODIPY scaffold upon BF_3_ post-condensation. Thus, GBBR adduct 16 was synthesised and its BODIPY-like analogue 17 suitably obtained, rendering a non-conventional 5-membered ring BODIPY derivative (Figure 7g). Although the GBBR adduct is somewhat fluorescent, the boron bridging greatly improves the photophysical properties. Probe 17 displays an impressive 60 nm red-shift and a 40-fold fluorescence increase (compared with 16) while being pH independent, making it a valuable fluorophore for bioimaging applications under a range of conditions (Figure 7h). Its potential in live-cell imaging was confirmed by incubation of compound 17 with human lung A549 epithelial cells, showing remarkable cell permeability and exhibiting preferential accumulation in mitochondria (Figure 7i).

**Figure 8:**
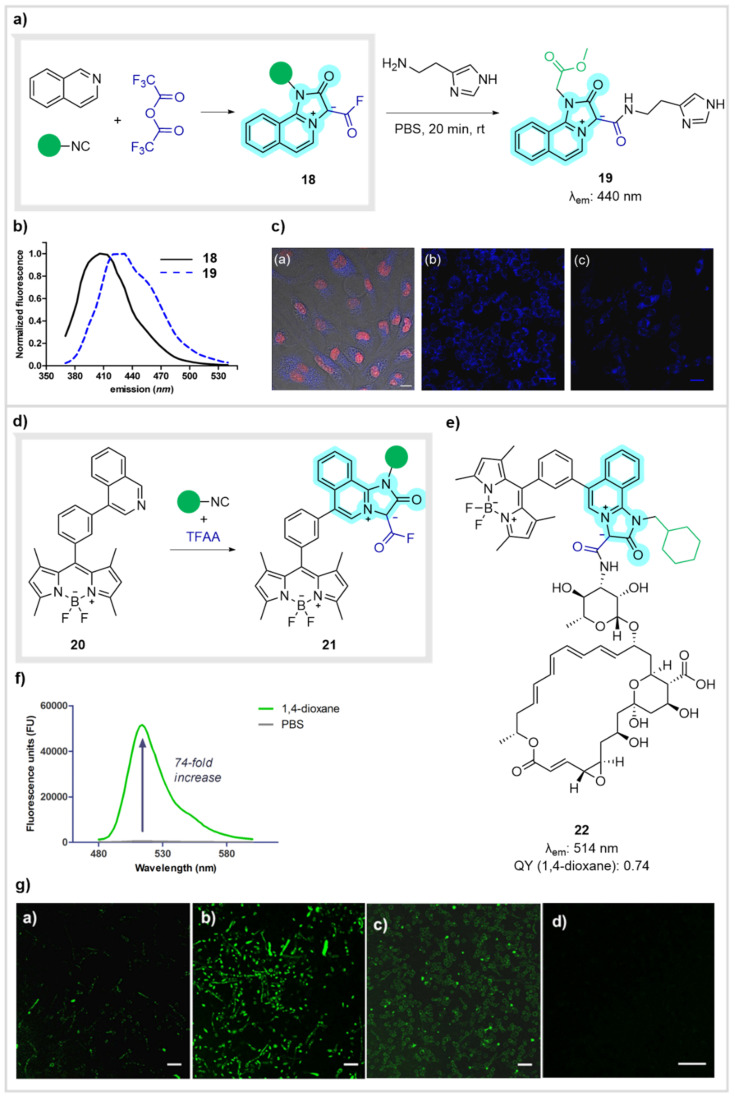
(**a**) MCR access to probe **18** for imaging of histamine in live cells. (**b**) Normalized fluorescence of probe **18** (black) and histamine adduct **19** (blue). (**c**) Microscopy images of: (**a**) RBL-2H3 basophils upon incubation of probe **18** with DRAQ5 as nuclear counterstaining; (**b**) RAW 264.7 macrophages after incubation with Histamine blue and upon histamine uptake and (**c**) after treatment with thapsigargin. Sections (b,c) from ref [48] (N. Kielland et al., *Chem. Commun*. **2012**, *48*, 7401) reproduced with permission form the Royal Society of Chemistry. (**d**) MCR to the BODIPY dipolar acid fluoride **21**. (**e**) Structure of probe **22**, from conjugation of **21** with natamycin. (**f**) Fluorescent properties of probe **22** in 1,4-dioxane and in PBS. (**g**) Fluorescence images of fungal and bacterial cells upon incubation with **20**: (**a**) *F. solani*, (**b**) *F. oxysporum*, (**c**) *A. flavus* and (**d**) *P. Aeruginosa*. Scale bar: 20 μm. Sections (**f**,**g**) from ref [50] (M. Sintes et al. *Bioconjug. Chem*. **2016**, *27*, 1340) reproduced with permission. Copyright 2016 ACS.

In the context of de novo synthesis of fluorescent scaffolds, we also described a new MCR from azines, isocyanides and trifluoroacetic acid anhydride that yielded mesoionic acid fluorides (Figure 8a) [48]. Their remarkable stability to hydrolysis together with their photochemical properties made this scaffold promising to detect amino containing analytes through amide formation. When we reacted adduct 18 with a series of signaling molecules, mainly hormones and neurotransmitters, in PBS solution, we found that it exhibited an excellent selectivity towards histamine. Moreover, a significant bathochromic shift was observed, and clear differences between the probe (named Histamine Blue) and the histamine adduct 19 could be observed (Figure 8b). Then, we evaluated its ability to image histamine in live RBL-2H3 basophils and RAW 264.7 macrophages. Histamine Blue (18) selectively stained the histamine-storing granules of basophils, without inducing cytotoxicity or affecting their morphology. As for the macrophages, probe 18 did not stain the cells, which under normal conditions contain low levels of histamine. However, macrophages were brightly stained after either histamine uptake or treatment with thapsigargin, which induces de novo synthesis of histamine (Figure 8c) [48].

Based on these results, we also tackled the preparation of new labelling agents by modifying our acid fluoride probe with well-established fluorophores, following facilitated procedures for sophisticated constructs [49]. We realized that linking the BODPIY scaffold to our mesoionic acid fluorides would potentially provide labels for amine-containing biomolecules endowed with remarkable properties. Indeed, when the BODIPY isoquinoline 20 took part in the MCR, it suitably yielded the corresponding dipolar acid fluoride 21 (Figure 8d) [50]. As expected, the probe maintained the spectral properties of the BODIPY dye as well as the resistance to hydrolysis and exclusive reactivity towards amines of the initial acid fluorides and validated it for use as a label. In this regard, we tackled the visual detection of natamycin through this approach. This macrolide selectively binds to ergosterol, a key component of fungal cell membranes, thus inhibiting their growth. It also contains a free amino group eligible for coupling with our probe. Thus, the fluorescent natamycin amide 22 could serve as a probe for the detection of fungal infection sites (Figure 8e). Moreover, this compound displayed remarkable environment sensitivity towards hydrophobic media, showing a 70-fold fluorescence increase in 1,4-dioxane (mimicking the lipid bilayer) over PBS (mimicking the intracellular media) (Figure 8f). Its use as a fluorescent probe for the imaging of fungal cells was assessed by incubation with several fungal pathogens (*Fusarium solani*, *F. oxysporum*, and *Aspergillus flavus*) and bacteria (*Pseudomonas aeruginosa*). While the ergosterol-containing fungi species displayed bright fluorescence upon uptake of the probe (detected by confocal microscopy); bacteria, lacking ergosterol, were not stained, confirming the potential application of BODIPY-natamycin derivative 22 as a fluorescent probe for imaging fungal infections (Figure 8g).

## 6. Receptors

This section describes the development of neuroactive agents via MCRs and related processes. These receptor modulators address symptomatic treatment for neurodegenerative diseases, aiming to improve cognitive and behavioral signs. This goal is very challenging, as no new cognitive enhanced drug has reached the market in more than two decades. In this context, we were attracted by imidazoline I_2_ receptors (I2-IR), located in central and peripheral nervous system, as well as many organs and tissues [51]. I_2_-IR are imidazoline nonadrenergic binding sites recognized by the tritiated radioligands idazoxan and *p*-aminoclonidine [52,53]. Although, I_2_-IR are heterogeneous and lack detailed structural definition, their implication in physiological and pathological processes has been described by using well-characterized I_2_-IR ligands. These ligands have proven that I_2_-IR level dysregulations are a hallmark in analgesia, inflammation, and human brain disorders such as AD, Parkinson’s and Huntington diseases, depression, and glial tumors [54,55,56].

**Figure 9:**
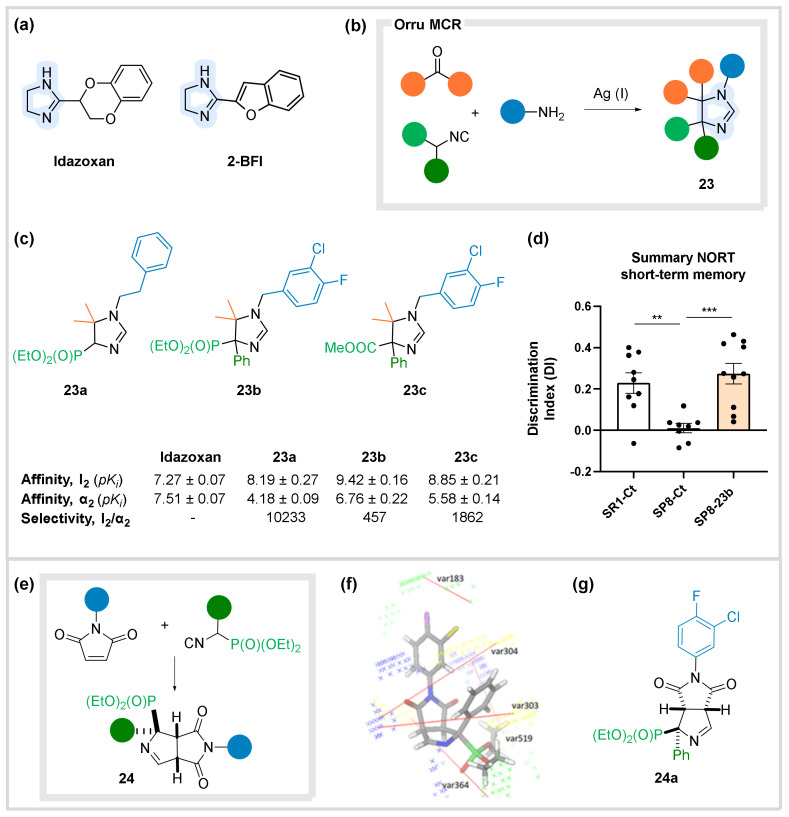
I_2_ Imidazoline receptors. (**a**) Structure of known I_2_IR ligands. (**b**) MCR access to substituted 2-imidazolines **23**. (**c**) Structure and binding affinities of the selected compounds. (**d**) In vivo data of compound **23b**. (**e**) Condensation between maleimides and PhosMIC derivatives leading to new I_2_-IR ligands **24**. (**f**) 3D-QSAR series **24**. (**g**) Structure of the selected analogue **24a**. Values represented in (**d**) are mean ± Standard error of the mean (SEM); n = 36 (SR1-Ct n = 11; SP8-Ct n = 11; SP8-23b n = 14). ** *p* < 0.01; *** *p* < 0.001.

Known I_2_-IR ligands share common structural features, namely a 2-substituted imidazoline (Figure 9a) [57,58,59]. As substitution in the 4 and 5-positions of the imidazoline ring had remained unexplored, we tackled these structural changes to generate new families of I_2_-IR ligands with enhanced pharmacological properties. To achieve a fast and convenient access to these imidazoline scaffolds, we applied the Orru MCR, which involves the use of carbonyls, amines and isocyanides bearing an acidic α-position (Figure 9b) [60,61]. We specifically studied the role of diethyl isocyanomethylphosphonate (PhosMic) in these processes and reported the silver-catalyzed MCR synthesis of a diverse library of 2-(imidazoline-4-yl)phosphonates **23** [62]. The pharmacological profile and selectivity of the selected compounds were evaluated through competition binding studies against the tritiated selective radioligands for I_2_-IR (2-BFI) and α_2_-adrenoreceptors (AR) such as 2-methoxyidazoxan. The studies were performed in membranes from postmortem human frontal cortex, a brain area with significant density of both receptors. Compounds **23a** and **23b** emerged as the most promising ligands. Imidazoline **23a** showcased a much better I_2_/α_2_- AR selectivity than the standard idazoxan, while analogue **23b** displayed remarkable nanomolar-range affinity for I_2_-IR (Figure 9c) [63].

With the impressive results of our new I_2_-IR ligands, we explored their anti-AD properties. Accordingly, in vivo studies in mice showed that acute treatments with MCR adducts **23a** and **23b** in the mice hippocampus significantly increased the ratio of oligomeric FAS-associated protein with death domain (FADD) phosphorylated at ser191 (p-FADD) to total dimeric FADD, an index of cell survival and neuroplasticity. Both compounds also induced mild hypothermia, a well-stablished neuroprotective effect in cerebral ischemia, regulated mechanisms of apoptotic pathways and inhibited p35 cleavage into neurotoxic p25 [63]. Moreover, in vivo studies in the model of neurodegeneration senescence accelerated mouse prone 8 mice (SAMP8) with compounds **23b** and **23c** (endowed with high affinity and selectivity for I_2_-IR) produced beneficial effects in behavior and cognition. Changes in molecular pathways implicated in oxidative stress, inflammation, synaptic plasticity, and apoptotic cell death supported the improvement in the whole condition of the treated animals. This study was the first experimental evidence to demonstrated that I_2_-IR are putative targets for cognitive impairment [64]. Therefore, I_2_-IR could be considered as a putative new therapeutic target for AD, and its modulation constitutes an encouraging option for treating this unmet medical need. Additionally, in vivo studies in SAMP8 animals treated with adduct **23b** ameliorated both behavioral and psychological symptoms of dementia and cognitive decline by attenuating depressive-like and fear-anxiety-like behavior and improving cognitive performance. Amelioration of molecular pathways underlying depression and anxiety phenotypes were observed, suggesting I_2_-IR as an alternative target for slowing down AD progression (Figure 9d) [65].

In parallel, we described a related process (not a MCR this time) that involved the interaction of a PhosMic component with a maleimide, to yield a formal [3 + 2] cycloadduct, displaying an alternative substitution pattern [66]. In this way, another chemset of 36 bicyclic adducts **24** was conveniently prepared (Figure 9e). These bicyclic adducts showed outstanding affinity and selectivity values upon I_2_-IR and constituted the first example of I_2_-IR ligands without a 2-imidazoline unit. Interestingly, 3D-QSAR studies with these series helped to rationalize the results and, circumventing the limited structural information of the receptor, guided the design of the future improved ligands (Figure 9f). In this way, the mapping of the receptor suggested the type and polarity of further substitutions in the scaffold. Preliminary ADME and a safety panel led us to select a representative compound **24a** (Figure 9g) for in vivo studies [67], which demonstrated hypothermic properties and neuroprotection in a murine model of neurodegeneration [68]. Additionally, the treatment of SAMP8 murine model with **24a** revealed a beneficial effect by improving cognition and ameliorating anxiety-like behavior. Modulation of I_2_-IR with compound **24** reduces neuroinflammation, oxidative stress and calcineurin protein levels in SAMP8 [69].

The suitable access to undescribed 2-(imidazoline-4-yl)phosphonate and related scaffolds, enabled by judicious use of MCRs, has increased the chemical diversity of the I_2_-IR modulating ligands and importantly unveiled their implication in neurodegenerative diseases. Further progress in the area will be supported by the robust chemistry developed ensuring the convenient preparation of the required ligands.

## 7. Conclusions and Outlook

The incorporation of MCRs to the synthetic arsenal in biomedical projects greatly empowers the targeted preparation of defined structures and combinatorial libraries, as well as enabling the exploration of the dark chemical space. It constitutes an attractive strategy, already delivering useful scaffolds and hits impossible to prepare otherwise. The development of new transformations belonging to this family of reactions is an ongoing task in several research groups and it will surely help in the colonization of these uncharted regions. The presented results dealing with directed synthesis based on rational design, combinatorial approaches and diversity-oriented (exploratory) preparations spanning a variety of targets including enzymes (AChE, DHFR), transcription factors (AhR), receptors (I2-IR), chemotherapeutical hits (viruses, bacteria) and fluorescent probes (for histamine, lysosomes, etc.), validate the hypothesis. In summary, we have developed rationally designed enzyme inhibitors, prepared through previously described MCRs, with unprecedented potency and selectivity. Moreover, in a sort of late-stage functionalization, we have deeply modified a useful antibiotic and improved its properties. The use of well-established MCRs also paved the way to access new classes of receptor ligands which were much improved through medicinal chemistry principles. We have synthesized a useful and diversifiable fluorophore precursor (BODIPY isocyanide) and generated useful bioprobes via standard MCRS. Finally, through reaction discovery, we have disclosed new chemotherapeutic compounds, labeling agents and transcription factor ligands. Future work along fundamental reactivity exploration and meaningful use of MCRs, mainly based in heterocycles, will likely increase the impact of the methodology and expand its reach in medicinal chemistry, encompassing all phases of the drug discovery and drug development.

## Data Availability

Data sharing not applicable.

## Abbreviations

AChE	Acetylcholinesterase
AD	Alzheimer disease
DDQ	2,3-Dichloro-5,6-dicyano-1,4-benzoquinone
BACE	Beta secretase
GBBR	Groebke-Blackburn-Bienaymé MCR
TMP	Trimethoprim
WHO	World Health Organization
MIC	Minimum inhibition concentration
PDB	Protein data bank
DHFR	Dihydrofolate reductase
SMX	Sulfamethoxazole
PaβN	Phenylalanine-arginine β-naphthylamide
NADPH	Reduced nicotinamide adenine dinucleotide phosphate
AhR	Hydrocarbon Receptor
FICZ	6-Formylindolo [3,2-b]carbazole
SAR	Structure-Activity Relationship
Trp	Tryptophan
TMSCl	Trimethylsilyl chloride
PBS	Phosphate buffer saline
AR	α_2_-Adrenoreceptors
PhosMic	Diethyl isocyanomethylphosphonate
